# Rice *TSV3* Encoding Obg-Like GTPase Protein Is Essential for Chloroplast Development During the Early Leaf Stage Under Cold Stress

**DOI:** 10.1534/g3.117.300249

**Published:** 2017-11-21

**Authors:** Dongzhi Lin, Quan Jiang, Xiaojing Ma, Kailun Zheng, Xiaodi Gong, Sheng Teng, Jianlong Xu, Yanjun Dong

**Affiliations:** * Development Center of Plant Germplasm Resources, College of Life and Environment Sciences, Shanghai Normal University, 200234, China; † Institute of Genetics and Developmental Biology, Chinese Academy of Sciences, Beijing 100101, China; ‡ Shanghai Institutes for Biological Sciences, Chinese Academy of Sciences, 200032, China; § The Institute of Crop Sciences, Chinese Academy of Agricultural Sciences, Beijing 100081, China

**Keywords:** chloroplast development, ribosome biogenesis, rice (*Oryza sativa* L.), Spo0B GTP-binding protein (Obg), thermo-sensitive virescent

## Abstract

The Spo0B-associated GTP-binding (Obg) proteins are essential for the viability of nearly all bacteria. However, the detailed roles of Obg proteins in higher plants have not yet been elucidated. In this study, we identified a novel rice (*Oryza sativa* L.) *thermo-sensitive virescent* mutant (*tsv3*) that displayed an albino phenotype at 20° before the three-leaf stage while being a normal green at 32° or even at 20° after the four-leaf stage. The mutant phenotype was consistent with altered chlorophyll content and chloroplast structure in leaves. Map-based cloning and complementation experiments showed that *TSV3* encoded a small GTP-binding protein. Subcellular localization studies revealed that TSV3 was localized to the chloroplasts. Expression of *TSV3* was high in leaves and weak or undetectable in other tissues, suggesting a tissue-specific expression of *TSV3*. In the *tsv3* mutant, expression levels of genes associated with the biogenesis of the chloroplast ribosome 50S subunit were severely decreased at the three-leaf stage under cold stress (20°), but could be recovered to normal levels at a higher temperature (32°). These observations suggest that the rice nuclear-encoded *TSV3* plays important roles in chloroplast development at the early leaf stage under cold stress.

The chloroplast is a semiautonomous organelle that contains many genes important for the metabolic pathways of photosynthesis (Mandel *et al.* 1996). Chloroplast development during leaf development consists of a series of processes associated with chloroplast differentiation, which can be divided into three steps coregulated by the plastid and nuclear genes (Mullet 1993; Kusumi *et al.* 2011). The first step involves the activation of plastid replication and plastid DNA synthesis. The second step is the chloroplast “build up,” characterized by the establishment of the chloroplast genetic system. At this step, nuclear-encoded plastid RNA polymerase (NEP) preferentially transcribes plastid genes encoding plastid gene expression machineries (Hajdukiewicz *et al.* 1997), and the transcription and translation activities in the chloroplast are dramatically increased. In the third step, the plastid and nuclear genes that encode the photosynthetic apparatus are expressed at very high levels. The plastid genes for the photosynthetic apparatus are predominantly transcribed by plastid-encoded RNA polymerase (PEP) (Santis-Maciossek *et al.* 1999). Expression of all of these genes leads to chloroplast biosynthesis and assembly. In spite of this basic understanding of chloroplast development, the mechanisms of the major genes involved in this process in higher plants remain largely unknown (Pfalz and Pfannschmidt 2012).

GTPases comprise a large family of enzymes that hydrolyze guanosine triphosphate (GTP). GTPases occur in all of the kingdoms of life and are known to play crucial roles in many cellular processes (Bourne *et al.* 1990). The Spo0B-associated GTP-binding (Obg) protein subfamily of GTPases was originally identified downstream of the Spo0B gene in *Bacillus subtilis* (Trach and Hoch 1989). Typical Obgs are large GTPases containing three domains: the Obg fold, the G domain, and the Obg C-terminal region (OCT) (Buglino *et al.* 2002; Kukimoto-Niino *et al.* 2004). The Obg proteins have been shown to be essential for the viability of nearly all bacteria (Maddock *et al.* 1997; Okamoto and Ochi 1998; Shepherd *et al.* 2002; Foti *et al.* 2005; Michel 2005). With the exception of the *Escherichia coli* homolog ObgE, which is involved in chromosome partitioning and is partially associated with the cell membrane (Kobayashi *et al.* 2001), the majority of the Obg proteins studied to date are associated with the ribosome. For example, Obgs from *B. subtilis*, *Caulobacter crescentus*, and *E. coli* have been reported to be associated with the 50S ribosomal subunit (Scott *et al.* 2000; Lin *et al.* 2004; Wout *et al.* 2004). Furthermore, mutations in the Obg proteins have been shown to affect ribosome assembly or maturation (Datta *et al.* 2004; Jiang *et al.* 2006). Recently, mutations in the Obg fold and the OCT region of *B. subtilis* Obg impaired its ability to associate with the ribosomes and to induce a stress response, suggesting that Obg plays dual functions in ribosome biogenesis and stress responses (Kuo *et al.* 2008). Among eukaryotes, an *Arabidopsis* Obg homolog, *At*ObgC/CPSAR1, which localizes both in the inner envelope and the stroma of chloroplasts, has been shown to be essential for the formation of thylakoid membranes (Garcia *et al.* 2010). However, others have suggested that *AtObgC/CPSAR1* may play an important role in the biogenesis of chloroplast ribosomes (Bang *et al.* 2009). More recently, studies on *Arabidopsis AtObgC/CPSAR1* and rice *OsObgC* led Bang *et al.* (2012) to conclude that ObgC functions primarily in plastid ribosome biogenesis during chloroplast development.

Rice (*Oryza sativa*) mutants are ideal for explicating the function of chloroplast development in higher plants (Sachetto-Martins *et al.* 2000; Leister 2003). In this study, we identified a new rice thermo-sensitive virescent mutant *tsv3*, which exhibited an albino phenotype before the three-leaf stage at 20° and a normal green color at 32° or even at 20° after the four-leaf stage. Mutation of rice *TSV3*, encoding the Obg subfamily of small GTP-binding protein, was responsible for the mutant phenotype. Additionally, the transcript levels of genes associated with chlorophyll (Chl) biosynthesis and photosynthesis, and those associated with biogenesis of the chloroplast ribosome 50S subunit, were severely affected in the *tsv3* mutants at low temperature. These findings suggest that rice *TSV3* plays an important role in chloroplast biogenesis during leaf development under cold stress.

## Materials and Methods

### Plant materials and growth conditions

The rice *thermo-sensitive virescent* mutant, *tsv3*, was discovered in our mutant pool from the *japonica* cv. Jiahua 1 [wild type (WT)] irradiated with ^60^Co gamma rays in 2006. The F_2_ population for genetic mapping was generated from a cross between the *indica* cv. Pei’ai 64S and the *tsv3* mutant. The thermo-sensitive virescent mutant phenotype of *tsv3* could be distinguished from the normal (WT) green phenotype during the winter season at Hainan, China (subtropical climate) and the spring season in Shanghai, China (temperate climate) under local growing conditions. WT and *tsv3* plants were grown in growth chambers under a controlled photoperiod (12 hr of light and 12 hr of dark) and at a constant temperature of either 20 or 32° for phenotypic characterization, pigment content measurement, and RNA extraction.

### Chl and carotenoid content measurement

Both Chl and carotenoid (Car) content were assessed using a spectrophotometer following the methods of Arnon (1949) and Wellburn (1994) with slight modifications. Briefly, 0.2 g of fresh leaves were harvested from the three-leaf-stage seedlings grown at 20 or 32° and were homogenized in a 5-ml solution of acetone:ethanol:H_2_O (5:4:1) for 18 hr under dark conditions. Residual debris was removed by centrifugation. The supernatants were analyzed using a UV5100 Spectrophotometer (Beckman Coulter) at 663, 645, and 470 nm.

### Transmission electron microscopy

The tops of the third leaves from the three-leaf-stage seedlings grown at 20 or 32° were used for preparing transverse sections for transmission electron microscopy (TEM) analysis. Leaf samples were fixed in a solution of 2.5% glutaraldehyde followed by 1% OsO_4_ buffer at 4° for 5 hr after vacuum. After staining with uranyl acetate, tissues were further dehydrated in an ethanol series and finally embedded in Spurr’s medium prior to ultrathin sectioning. Samples were stained again and examined under a Hitachi (Tokyo, Japan) H-7650 Transmission Electron Microscope.

### Mapping of the TSV3 gene

Rice genomic DNA was extracted from fresh leaves using the modified CTAB method (Murray and Thompson 1980). A total of 1430 plants with the mutant phenotype were selected from the F_2_ populations. Initially, we used 81 SSR primers based on the Gramene database (http://www.gramene.org). New SSR and InDel markers were developed later based on the genomic sequences available for *japonica* cv. Nipponbare (Goff *et al.* 2002) and *indica* cv. 9311 (Yu *et al.* 2002). Details of the markers used for mapping are listed in Supplemental Material, Table S1. Genomic DNA fragments of the candidate genes from the mutant and WT plants were PCR amplified and sequenced (SinoGenoMax, Shanghai, China). The function and open reading frames of the candidate genes were obtained from TIGR (http://rice.plantbiology.msu.edu/cgi-bin/gbrowse/rice/). Conserved domain structures were predicted using SMART (http://smart.embl-heidelberg.de/).

### RT-PCR and real-time PCR analyses

Expression of *TSV3* was monitored in various tissues of Jiahua 1 (WT) plants including germinating buds, plumules, roots, stems, and young leaves before the five-leaf stage, and flag leaves and young panicles at the heading stage. Total RNA was isolated using an RNA Prep Pure Plant kit (TIANGEN, Beijing, China), reverse transcribed using ReverTra Ace (ToYoBo, Osaka, Japan) following the manufacturer’s instructions, and subjected to RT-PCR analysis.

To quantify *TSV3* expression level, real-time PCR (qPCR) analyses were performed. An additional set of 23 other genes associated with Chl biosynthesis, chloroplast development, and photosynthesis in rice were subjected to qPCR (*HEMA1*, *CAO1*, *YGL1*, *PORA*, *Cab1R*, *RbcS*, *RbcL*, *PsaA*, *PsbA*, *LhcpII*, *RNRS*, *RNRL*, *V2*, *OsRpoTp*, *OsPoLP1*, *FtsZ*, *RpoA*, *RpoB*, *Rps7*, *Rps20*, *Rpl21*, *16SrRNA*, and *23SrRNA*). The rice *Actin* gene was used as a reference. Total RNA was extracted from the third leaves of WT and *tsv3* plants. The qPCR analyses were performed using the SYBR Premix Ex TaqTM kit (TaKaRa, Tokyo, Japan) on an ABI7500 Real-Time PCR System (Applied Biosystems; http://www.appliedbiosystems.com). Relative gene expression was quantified as described previously (Livak and Schmittgen 2001). Primers used for qPCR were designed according to both Wu *et al.* (2007) and NCBI-published sequences (Table S2).

### Complementation test

An 8.3-kb genomic DNA fragment spanning the entire *TSV3* gene, plus an additional 2.0-kb sequence upstream and downstream of *TSV3*, was PCR amplified from the WT parent using gene-specific primers (*TSV3*F: 5′-GGGGTACCCCTTGACATACCTCTCCTGTTTGC-3′ and *TSV3*R: 5′-CGGGATCCCGCTGGGTTGGACAGATAATATGC-3′, where the underlined sequences represent the *Kpn*I and *BamH*I restriction sites, respectively). The PCR product was cloned into the pMD18-T vector (TaKaRa, Tokyo, Japan), and the fragment was cloned into the pCAMBIA1301 binary vector (CAMBIA; http://www.cambia.org.au) after sequence verification. The resultant pCAMBIA1301-TSV3 plasmid and the empty vector (control) were introduced into *Agrobacterium tumefaciens* EHA105 and transformed into the *tsv3* mutant via *Agrobacterium*-mediated transformation (Hiei *et al.* 1994). Transgenic plants were identified by PCR amplification of the *hygromycin phosphotransferase* (*hpt*) gene (*HPT*F: 5**′**-GGAGCATATACGCCCGGAGT-3**′** and *HPT*R: 5**′**-GTTTATCGGCACTTTGCATCG-3**′**) and β*-glucuronidase* (*GUS*) gene (*GUS*F: 5**′**-GGGATCCATCGCAGCGTAATG-3**′** and *GUS*R: 5**′**-GCCGACAGCAGCAGTTTCATC-3**′**).

### Subcellular localization

To investigate the subcellular localization of TSV3 protein, a cDNA fragment containing the N-terminal region of *TSV3* (1–280 aa) was amplified from total RNA in WT plants using the primer pair 5**′**-GAAGATCTATGCCGCTCCTCCTCCAC-3**′** and 5**′**-GGGGTACCCCACCAACGTCTGCAACCAC-3**′** (where the underlined sequences represent *Bgl*II and *Kpn*I restriction sites, respectively), and introduced into the pMON530-GFP vector. The resulting pMON530:CaMV*35S:TSV3-GFP* plasmid was sequence confirmed and introduced into the *Agrobacterium* strain EHA105. For subcellular localization of TSV3, transient expression assays were performed in tobacco (*Nicotiana tabacum*) as described (Jiang *et al.* 2014). The GFP fluorescence images were obtained using an argon ion laser excitation of 488 nm with a 505- to 530-nm band pass filter.

### Sequence and phylogenetic analyses

Gene prediction was performed using the Rice Genome Annotation Project (RGAP) database (http://rice.plantbiology.msu.edu/). The full-length amino acid sequence of *TSV3* was used to identify orthologs using a BLASTP search. The orthologous protein sequences were aligned using the MUSCLE tool (Edgar 2004) set at default parameters. A neighbor-joining tree was constructed with MEGA v5.2 software (http://www.megasoftware.net/; Tamura *et al.* 2011) using the bootstrap method with 1000 replicates. Multiple sequence alignments were conducted using BioEdit software (http://www.mbio.ncsu.edu/BioEdit/bioedit.html; Hall 1999).

### Data availability

The authors state that all data necessary for confirming the conclusions presented in the article are represented fully within the article.

## Results

### Phenotypic characterization of the tsv3 mutant

**Figure 1:**
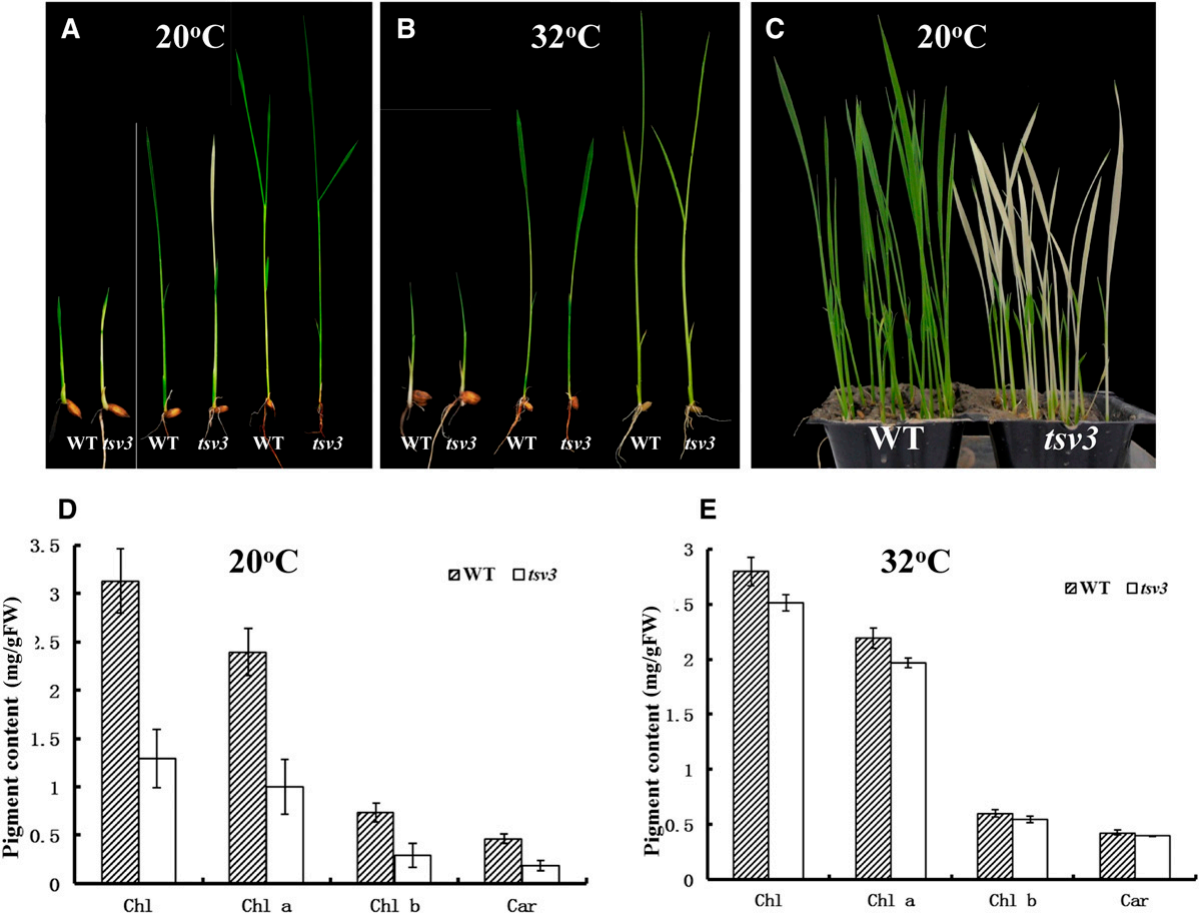
Characterization of the *tsv3* mutants. Seedlings of Jiahua 1 (WT, left) and *tsv3* mutant (right) at the two-, three-, and four-leaf stage grown at (A) 20° and at (B) 32°. (C) Three-leaf-stage seedlings for WT (left) and *tsv3* mutant (right) grown at 20°. Measurements of total Chl, Chl a, Chl b, and Car contents of the third leaf harvested from the three-leaf-stage seedlings of WT and *tsv3* mutants grown at (D) 20° and (E) 32°. Pigment contents are expressed in milligrams per gram fresh weight of leaf tissue.

Leaves of the *tsv3* mutant appeared albino only before the four-leaf stage when grown at 20° (Figure 1, A and C), gradually turned yellowish green, and finally a normal green after the four-leaf stage at 20° or a higher temperature (32°) (Figure 1B). Consistent with the phenotype, Chl a, Chl b, and Car contents of the third leaves of the *tsv3* mutant were drastically lower than those in the WT plants at 20° (Figure 1D), but were comparable to the WT and *tsv3* plants at 32° (Figure 1E). These observations suggested that the virescent phenotype of the *tsv3* mutant was thermo-sensitive at the early seedling stage.

**Figure 2:**
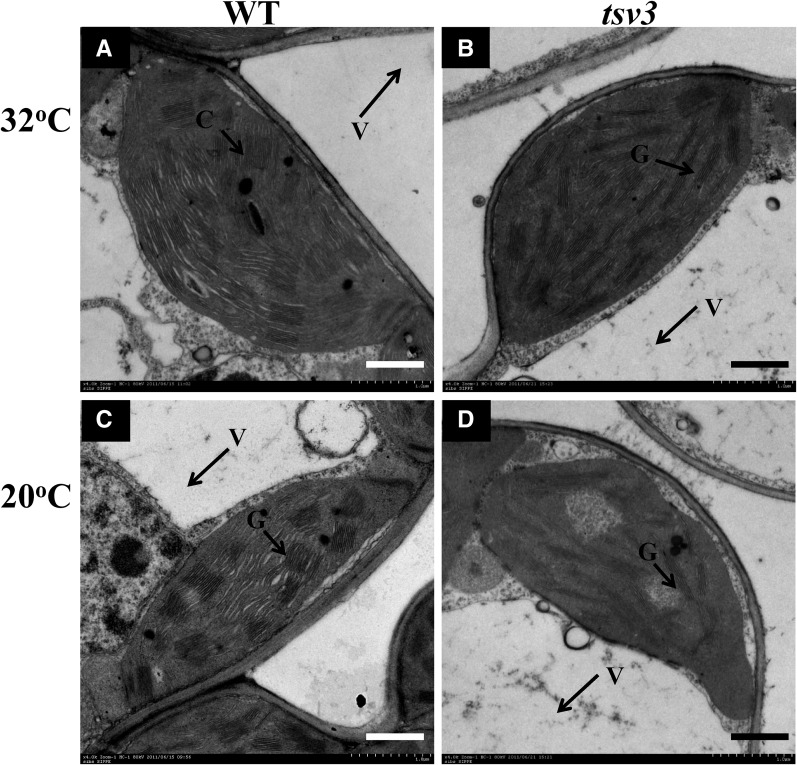
TEM images of chloroplasts in the WT and *tsv3* mutant plants grown at (A and B) 32° and (C and D) 20°. G, grana stacks; V, vacuole.

To examine whether the lack of photosynthetic pigments in the *tsv3* mutant at low temperatures was accompanied by ultrastructural changes in the chloroplast, the tops of the third leaves from the three-leaf-stage seedlings were analyzed using TEM. The grana lamella stacks in the WT plants were dense and well structured, regardless of the temperature at which the plants were grown (Figure 2, A and C). The *tsv3* mutant plants, on the other hand, exhibited abnormal chloroplast structure with fewer grana lamella stacks at 20° (Figure 2D). This abnormal phenotype of the *tsv3* mutants was corrected at a warmer temperature of 32°, where the chloroplasts displayed well-developed lamella structures (Figure 2B) similar to WT (Figure 2, A and C), suggesting the *tsv3* mutation affected chloroplast biogenesis only before the four-leaf stage under cold stress (20°). Moreover, except for the slight reduction in plant height of the WT plants after transplanting (Figure S1A), other yield-related traits such as panicle number, grains per panicle, and 1000-grain weight showed no significant differences between the WT and *tsv3* mutant plants (Figure S1B). This indicates that the *tsv3* mutation would not have any negative effects on plant growth under field condition.

### Identification of the gene underlying the tsv3 mutant phenotype

**Figure 3:**
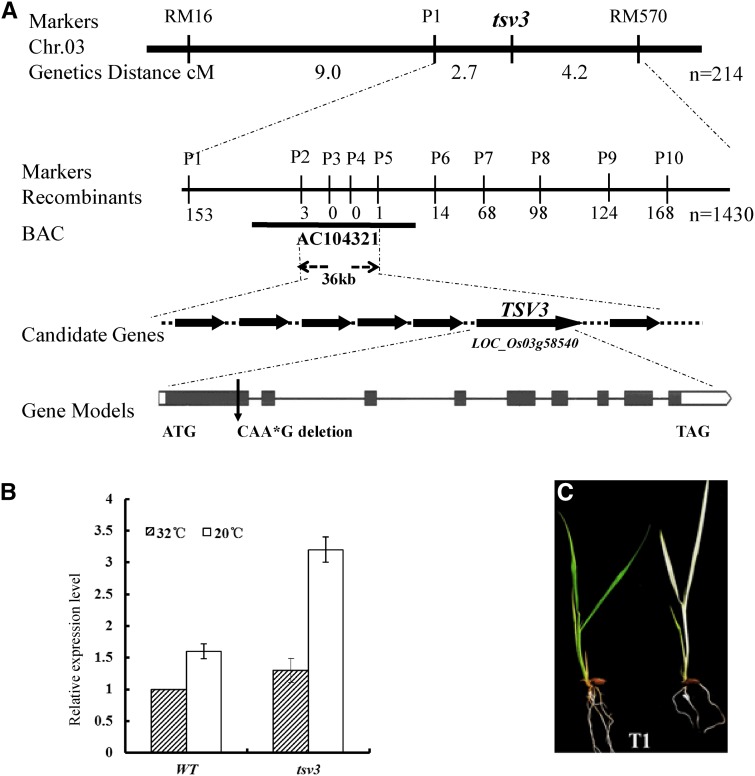
Mapping and genetic analysis of the *TSV3* gene. (A) Genetic mapping of the *TSV3* gene. (B) Relative expression level of *TSV3* (*LOC_Os03g58540*) in the mutant and WT seedlings at the three-leaf stage grown at 32 or 20°; *OsActin* was used as a control for qPCR. (C) The T_1_ segregation from transgenic T_0_ plants transformed with pCAMBIA1301-TSV3 at 20°. Phenotypes of T_1_ progeny: heterozygous mutant (*TSV3*/*tsv3*) with a normal green phenotype (left) *vs.* a homozygous mutant (*tsv3*/tsv3) with an albino phenotype (right).

To understand the molecular mechanism underlying the *tsv3* mutant phenotype, a map-based cloning strategy was used. The *indica* cv. Pei’ai 64S was crossed to the *tsv3* mutant. All plants of the F_1_ progeny displayed a normal green phenotype, while individuals of the F_2_ population displayed a 3:1 segregation ratio (green:albino phenotype of 453:132; χ^2^ = 3.45; *P* > 0.05), demonstrating that the albino phenotype is a recessive trait controlled by a single gene (*tsv3*). Using the PCR-based markers P1 and RM570 (Table S1) and 214 mutant F_2_ individuals, the *tsv3* locus was mapped to chromosome 3 (Figure 3A). Fine mapping using additional markers narrowed the *tsv3* locus down to a 36-kb interval between the markers P2 and P5 on the BAC clone AP104321; no recombinant was found between the markers P3 and P4 (Figure 3A and Table S1). Within this target region, seven candidate genes were predicted using the program RGAP (http://rice.plantbiology.msu.edu) and verified by sequencing. Four discontinuous nucleotide deletions (CAA*G) in the *tsv3* mutant were identified in the first exon of *LOC_Os03g58540*, which encodes an Obg subfamily of small GTP-binding protein (Figure 3A), causing a premature stop codon and consequently a frame-shift mutation. In addition, the significant upregulation of *LOC_Os03g58540* transcript in the *tsv3* mutant compared to the WT plants at 20° (Figure 3B) further suggested *LOC_Os03g58540* as the most probable gene underlying the *tsv3* locus.

To further confirm that the *LOC_Os03g58540* mutation was responsible for the *tsv3* mutant phenotype, a genetic complementation test was performed. The pCAMBIA1301 vector expressing *LOC_Os03g58540* under the control of its own promoter was introduced into the *tsv3* mutant plants. A total of 23 independent transgenic events were identified, all of which showed a complete reversion to the green leaf phenotype, comparable to the WT plants. Furthermore, the T_1_ progeny showed the segregation of mutant plants at 20° (Figure 3C). These data affirmed *LOC_Os03g58540* as the gene underlying the *TSV3* locus.

### Characterization of the TSV3 protein

TSV3 was predicted to be a 504-aa polypeptide with a calculated molecular mass of 54.5 kDa. Conserved domain analysis using pfam (http://pfam.janelia.org) showed that TSV3 encoded a Spo0B-associated GTP-binding protein (the Obg subfamily of small GTP-binding protein) containing a 50S ribosomal subunit-binding domain.

**Figure 4:**
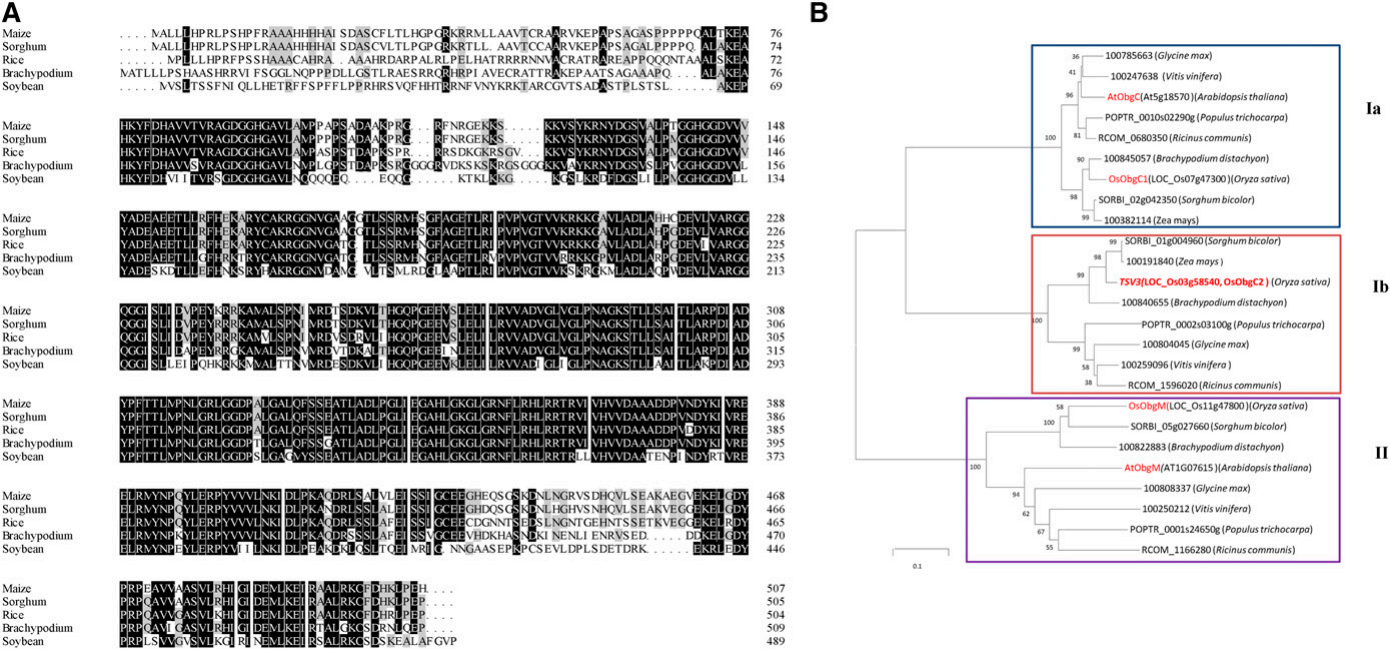
Phylogenic analysis of TSV3. (A) Amino acid sequence alignment of TSV3 orthologs of rice, maize, sorghum, *Brachypodium*, and soybean. Fully or partially conserved amino acids are shaded in black and gray, respectively. (B) Phylogenetic tree derived from analysis of rice TSV3 with eight orthologs. The rooted tree is based on a multiple sequence alignment generated using MEGA 5.2. Scale represents percent substitutions per site. Statistical support for the nodes is indicated.

Orthologs of rice TSV3 were identified in *Arabidopsis thaliana*, *Populus trichocarpa*, *Ricinus communis*, *Vitis vinifera*, *Brachypodium*, *Glycine max*, *Sorghum bicolor*, and *Zea mays*. The TSV3 sequences were highly conserved within higher plants. Rice TSV3 exhibited the highest amino acid sequence similarity with the TSV3 from *Z. mays* (84.9%), followed by *S. bicolor* (83.3%) (Figure 4A). Phylogenetic analysis clustered the TSV3 orthologs into two groups (I and II), with two subgroups within group I (Ia and Ib) (Figure 4B). Interestingly, the Ib group containing the TSV1 can be clearly divided into monocots and dicotyledons. Notably, the existence of three rice homologous Obg genes, TSV3, formerly termed as OsObgC2 (Bang *et al.* 2009), OsObgC1 (*LOC_Os07g47300*; Bang *et al.* 2009, 2012), and OsObgM (*LOC_Os 11g47800;* Bang *et al.* 2009) were previously reported. The predicted three-dimensional structure of TSV3 (Ib subgroup, OsObgC2) more closely resembled that of OsObgM and AtObgM (group II) than OsObgC1 (Ia subgroup) (Figure S2).

### Subcellular localization of TSV3

**Figure 5:**
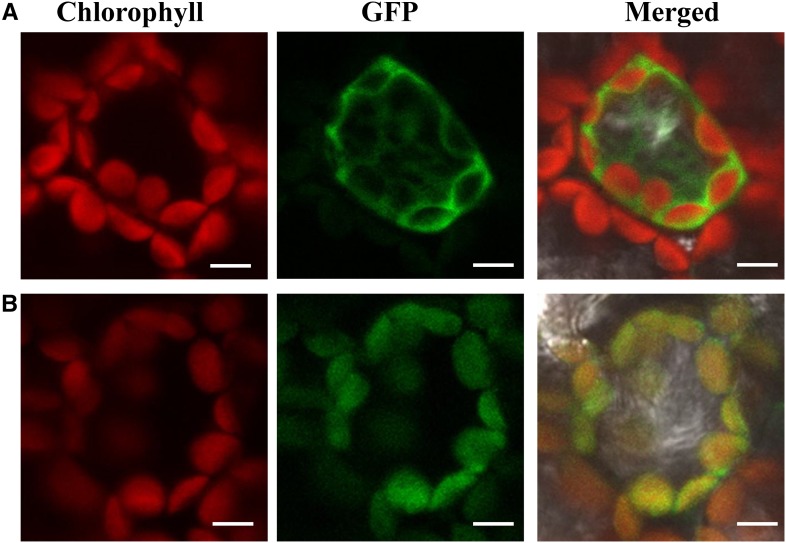
Subcellular localization of TSV3 protein. Tobacco cells transformed with (A) empty GFP vector without a specific targeting sequence and (B) TSV3-GFP fusion. Bar, 20 μm.

The protein subcellular localization prediction tools, TargetP (Emanuelsson *et al.* 2000; http://www.cbs.dtu.dk/services/TargetP/) and iPSORT (http://ipsort.hgc.jp/), predicted TSV3 to be localized in the chloroplasts. To verify this prediction, the pMON530:CaMV*35S:TSV3-GFP* plasmid was introduced into tobacco cells in a transient expression assay. The pMON530:*CaMV35-GFP* vector was used as a control. The GFP fluorescence signal in the tobacco mesophyll cells transformed with the pMON530:CaMV35S:TSV3-GFP plasmid perfectly overlapped with the chloroplast autofluorescence (Figure 5B), while the empty GFP vector without a specific targeting sequence showed green fluorescence in both the cytoplasm and the nucleus (Figure 5A). These results confirmed that TSV3 is localized in the chloroplast.

### Expression analysis of TSV3

**Figure 6:**
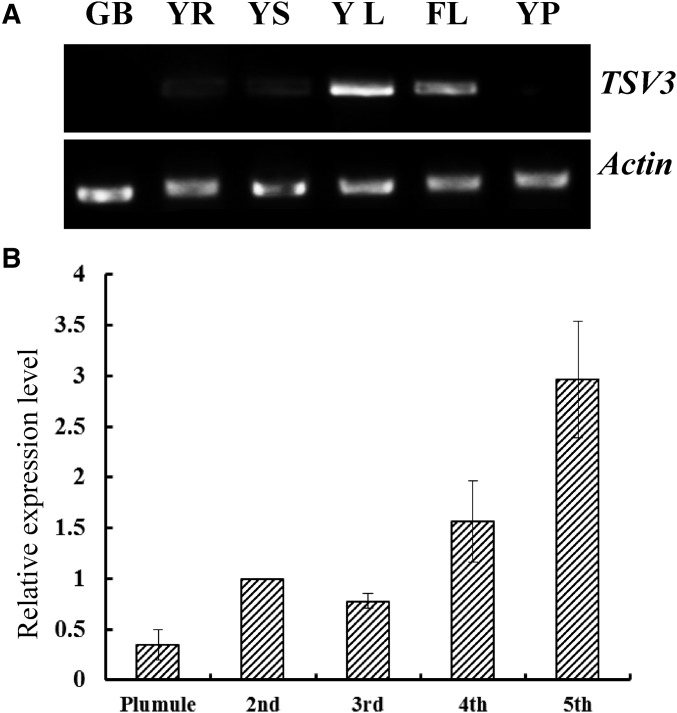
RT-PCR analysis of *TSV3*. (A) Expression of *TSV3* relative to *OsActin* in germinating buds (GB), young seedling roots (YR), young seedling stems (YS), young seedling leaves (YL), flag leaf at heading (FL), and young panicles (YP). (B) Transcript levels of *TSV3* relative to *OsActin* in the top-most leaves sampled from the plumule, and seedlings at the two-, three-, four-, and five-leaf stage. Relative *TSV3* transcript levels at all stages were normalized to the two-leaf stage.

To examine the expression pattern of *TSV3*, RT-PCR was performed on various tissues of WT plants, including germinating buds, plumules, roots, stems, and young leaves before five-leaf stage, and flag leaves and young panicles at the heading stage. Among all tissues examined, expression level of *TSV3* was highest in the leaves, with the young seedling leaves showing greater expression than the flag leaves (Figure 6A). The expression in the roots and stems of young seedlings was considerably lower and was almost undetectable in the germinating buds and young panicles (Figure 6A). This was consistent with the data from rice gene expression profiling in the RiceXPro database (Figure S3). In addition, the expression level of *TSV3* increased along with leaf development from the plumule to the five-leaf stage (Figure 6B). These results together with the phenotypic characterization of the *tsv3* mutants (Figure 1 and Figure 2) suggest that *TSV3* might play an important role in leaf chloroplast development, especially during the early seedling stage.

### TSV3 regulates the expression of chloroplast-associated genes

**Figure 7:**
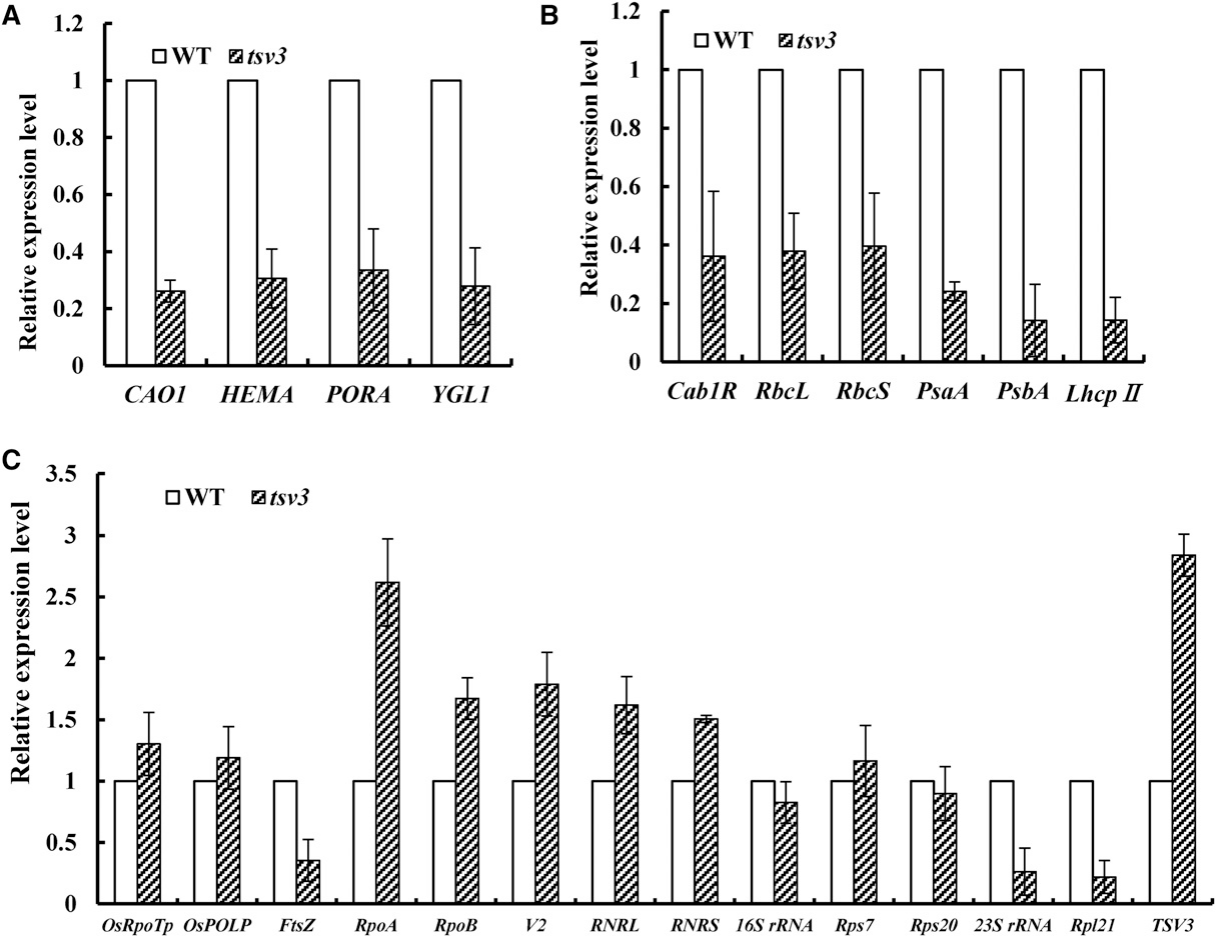
Quantitative expression analysis of genes related to (A) Chl biosynthesis, (B) photosynthesis, and (C) chloroplast development in the *tsv3* mutant and WT plants at 20°. Expression level of each gene was analyzed by qPCR and normalized relative to *OsActin*. Error bars represent SD (*n* = 3).

**Figure 8:**
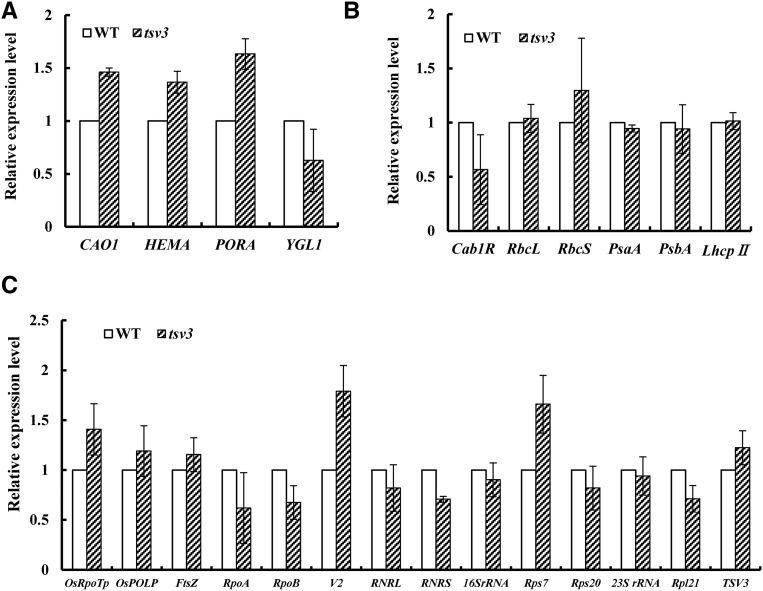
Quantitative expression analysis of genes related to (A) Chl biosynthesis, (B) photosynthesis, and (C) chloroplast development in the *tsv3* mutant and WT plants at 32°. Expression level of each gene was analyzed by qPCR and normalized relative to *OsActin*. Error bars represent SD (*n* = 3).

The transcript levels of genes involved in Chl biosynthesis, photosynthesis, and chloroplast development were examined in the *tsv3* mutant and WT plants at 20 and 32°. Genes involved in Chl biosynthesis (Wu *et al.* 2007); including *CAO1* (*CHLOROPHYLLIDE A OXYGENASE1*), *HEMA1* (encoding glutamyl tRNA reductase), *YGL1* (encoding a Chl synthetase), and *PORA* (encoding NADPH-dependent protochlorophyllide oxidoreductase); were downregulated in the *tsv3* mutant at 20° (Figure 7A), consistent with its decreased Chl content (Figure 1D) and albino phenotype (Figure 1, A and C). Genes associated with photosynthesis, including the plastid genes *Cab1R* (encoding the light harvesting Chla/b-binding protein of PSII), *PsaA* and *PsbA* (encoding two reaction center polypeptides), *RbcL* (encoding the large subunit of Rubisco), and the nuclear gene *RbcS* (encoding the small subunit of Rubisco) (Kyozuka *et al.* 1993), were greatly suppressed in the mutant at 20° (Figure 7B). However, at 32°, transcript levels of nearly all the above-mentioned affected genes were recovered to WT or slightly higher levels (Figure 8, A and B).

Among the genes associated with chloroplast development, we investigated nuclear-encoded genes such as *RNRS* and *RNRL* (encoding the large and small subunits of ribonucleotide reductase; Yoo *et al.* 2009); *V2* (encoding plastidial guanylate kinase; Sugimoto *et al.* 2007); *OsRpoTp* (encoding NEP core subunits; Hiratsuka *et al.* 1989); *OsPoLP1* (encoding one plastidial DNA polymerase; Vitha *et al.* 2001); *Rpl21* (encoding the ribosomal protein L21); *FtsZ* (encoding a component of the plastid division machinery; Takeuchi *et al.* 2007); and plastid-encoded genes including 23S ribosomal RNA (rRNA), 16S rRNA, *Rps7* (encoding one ribosomal protein; Kusumi *et al.* 2011), *Rps20* (encoding ribosomal protein S20; Gong *et al.* 2013), and *RpoA* and *RpoB* (encoding the PEP core α and β subunits; respectively; Kusumi *et al.* 2011). Relative expression levels of *FtsZ*, *23S rRNA*, and *Rpl21* were remarkably reduced in the *tsv3* mutant, while the other genes displayed WT levels or slightly higher levels at 20° (Figure 7C). Interestingly, all affected genes were recovered to WT levels at 32° (Figure 8C). It is possible that the abnormal expression of these key genes (*FtsZ*, *23S rRNA*, and *Rpl21*) led to the mutant phenotype under cold stress.

## Discussion

In this study, we cloned a novel rice gene, *TSV3*, which encoded a small GTP-binding protein of the Obg subfamily, using a map-based cloning strategy. Deletion of four nucleotides in the N-terminal region of *TSV3* resulted in a thermo-sensitive virescent phenotype and drastically affected the expression levels of certain genes associated with Chl biosynthesis, photosynthesis, and chloroplast development at low temperature (Figure 7).

### TSV3 is needed for early chloroplast development under cold stress

At a low temperature (20°), expression levels of genes associated with Chl biosynthesis and photosynthesis (Figure 7, A and B) as well as those of a few key genes involved in chloroplast development, such as *FtsZ*, *Rpl21*, and *23SrRNA*, were greatly reduced (Figure 7C) in the *tsv3* mutant compared to the WT. However, increasing the temperature to 32° upregulated the expression of these genes to levels that were either comparable or slightly higher than the WT plants (Figure 8). Additionally, the *tsv3* mutants exhibited an albino phenotype and abnormal chloroplasts at 20° before the three-leaf stage. Both of these phenotypes were corrected either with continued plant development (Figure 1) or with an increase in temperature (Figure 2). Together, these data suggested that *TSV3* was essential for early chloroplast development under cold stress. The finding that abnormal chloroplasts occur only in the early leaves of rice *tsv3* mutants under cold stress suggests that the function of TSV3 may not be a prerequisite at higher temperatures. Regarding the recovery of chloroplast development after the four-leaf stage under cold stress, TSV3 paralogs might perform the same or similar function. In addition, the *FtsZ* gene is known to be involved in the first step of chloroplast development (Takeuchi *et al.* 2007) and a reduced expression level of *FtsZ* in the *tsv3* mutant under cold stress (20°) might affect plastid division, which was consistent with the chloroplast development in the mutant (data not shown).

### TSV3 may regulate biogenesis of the chloroplast 50S large ribosomal subunit under cold stress

The chloroplast 50S large subunit consists of three rRNAs (23S, 4.5S, and 5S) and 30S ribosomal proteins, such as RPL21. Although much work has been undertaken to elucidate the composition of the chloroplast ribosome, the molecular basis of its assembly in higher plants remains elusive. Previous studies on bacteria showed that the majority of Obg proteins are associated with the 50S ribosomal subunit, ribosome assembly, and stress responses (Scott *et al.* 2000; Datta *et al.* 2004; Lin *et al.* 2004; Wout *et al.* 2004; Jiang *et al.* 2006; Kuo *et al.* 2008). In view of these results, rice *TSV3* may have similar functions in higher plants. Except for the OCT region, the Obg homologs in *Arabidopsis*, *At*ObgC/CPSAR1 (At5g18570), and in rice, *Os*ObgC1 (*LOC_Os07g47300*), share high structural similarity (Figure S2). *At*ObgC/CPSAR1 was shown to be essential for the formation of normal thylakoid membranes (Garcia *et al.* 2010) and was suggested to play an important role in the biogenesis of chloroplast ribosomes (Bang *et al.* 2009). More recently, based on the results of previous studies on rice *OsObgC1* (*LOC_Os07g47300*) and *Arabidopsis AtObgC* (*At5g18570*), Bang *et al.* (2012) reported that plant *ObgC* is a light-induced gene and its protein is translocated to chloroplast where it may be involved in the biogenesis of the large (50S) ribosomal subunit, thus influencing the PEP-related plastid gene transcription. The authors also proposed a hypothetical model of three ObgC domains (OCT, Obg fold, and G domain) resulting in ObgC-mediated regulation of chloroplast ppGpp signaling, association of ObgC with the 50S ribosomal subunit, and the action of ObgC depending on its GTP-/GDP-bound states, respectively (Figure S2).

In this study, *Rpl21* and *23SrRNA* genes encoding components of the chloroplast large ribosomal subunit (50S) were greatly downregulated in the *tsv3* mutant at 20° (Figure 7C); but the expression levels of genes encoding components of the chloroplast small ribosomal subunit (30S), including *Rps7*, *Rps20*, and *16SrRNA*, did not appear to be affected by temperature (Figure 7C and Figure 8C). Additionally, the severely reduced transcription levels of PEP-dependent plastid genes (*RbcL*, *psaA*, and *psbA*) at 20° (Figure 7B) suggested that the *tsv3* mutation affected the PEP transcription. These data suggest that, like *AtObgC* (Bang *et al.* 2012), rice *TSV3* might be involved in biogenesis of the large (50S), but not the small (30S), subunit of chloroplast ribosomes. This hypothesis was further supported by RNA gel blot analysis data, where the accumulation of the mature 23S rRNA was greatly reduced in the *tsv3* mutant only under cold stress, but nearly reached WT levels at high temperatures (Figure S3). Interestingly, only under cold stress, *TSV3* affected the 50S ribosome assembly and, in turn, produced the albino phenotype. Accordingly, the loss of TSV3-mediated activation of the *Rpl21-23S rRNA* mRNA regulation might produce a thermo-sensitive virescent phenotype under cold stress (Figure S4 and File S1).

As in the case of *AtObgC* RNAi *Arabidopsis* lines exhibiting chlorotic phenotypes, null mutants (*obgc1-d1* and *obgc1-t*) and the *OsObgC1* knockdown mutant (*obgc1-d2*) of *OsObgC1* (*LOC_Os07g47300*) in rice were reported to exhibit a severe or partially chlorotic phenotype during early leaf development regardless of the temperature (Bang *et al.* 2012). Additionally, the *OsObgC1* mutation has little effect on the expression of other rice Obg homologs such as *OsObgC2/TSV3* and *OsObgM* (Bang *et al.* 2009). In addition to its structural similarity to both OsObgM and AtObgM in mitochondria, the obvious differences between TSV3*/OsObgC2* and AtObgC1/OsObgC1 in their OCT regions (Figure S2) and their clustering in distinct subgroups of group I (Figure 4B) strongly support the existence of differences in their response to environmental changes (*e.g.*, temperature *vs.* light) and regulating pathways. These findings highlight the notion that even highly conserved genes within or across species might play more diverse and complex roles than previously recognized. Also, our observations provide evidence for versatile roles for ObgCs in plant development.

### TSV3 may be important for recovery from cold stress

In this study, the *tsv3* mutant is a typical thermo-sensitive virescent mutant, similar to the previously described thermo-sensitive virescent/albino rice mutants such as *v1* (Kusumi *et al.* 1997), *v2* (Sugimoto *et al.* 2004, 2007), *v3*, *st1* (Yoo *et al.* 2009), *wlp1* (Song *et al.* 2014), *osv4* (Gong *et al.* 2014), and *tcd9* (Jiang *et al.* 2014). Despite the similar phenotypes in *v1*, *v2*, *v3*, *st1*, *wlp1*, *osv4*, and *tcd9* mutants, the mechanisms and regulations affecting the chloroplast development are possibly different. Briefly, the *V1 (NUS1)* gene encoding the chloroplast-localized protein NUS1 regulates rRNA transcription under low temperature (Kusumi *et al.* 2011), and the *v1* mutation severely blocks the accumulation of PEP subunits (Kusumi *et al.* 2004). The *V2* gene, which encodes plastid/mitochondrial guanylate kinase, regulates guanine nucleotide pools (Sugimoto *et al.* 2007) under low temperature, and the *v2* mutation blocks the formation of functional PEP (Sugimoto *et al.* 2004). The *V3* and *St1* genes, encoding the large and small subunits of ribonucleotide reductase (RNR), respectively, are required for chloroplast biogenesis during early leaf development; and the *v3* and *st1* mutants withered to death at ∼30 d after germination at 20° (Yoo *et al.* 2009). In the *wlp1* mutant, the mutation of the rice large subunit protein L13 led to abnormal chloroplast development only under cold stress (Song *et al.* 2014). Additionally, mutation of rice *TCD9*, encoding the α subunit of chaperonin protein 60 (Cpn60α), hinders *FtsZ* transcription/translation and, in turn, influences plastid division and finally leads to abnormal chloroplasts under cold stress (Jiang *et al.* 2014). The mutation of *OsV4*, encoding a novel chloroplast-targeted PPR protein, leads to dramatically reduced transcription levels of some ribosomal components and PEP-dependent genes under cold stress (Gong *et al.* 2014).

Clearly, the loss of *TSV3* function produced the low-temperature-sensitive virescent phenotype before the four-leaf stage, indicating that *TSV3* was involved in a pathway that may be required only under cold stress. This was strongly supported by the high expression level of *TSV3* at 20°, regardless of the genotype (WT or mutant) (Figure 3B). It has been reported that cold stress disrupts protein biosynthesis in plastids by delaying translational elongation (Grennan and Ort 2007), and virescence/thermo-sensitivity are important for protection against photo-oxidative damage before healthy chloroplasts are developed (Zhou *et al.* 2009). Previous reports also confirmed that the deficiency of plastid translation often leads to a cold-sensitive phenotype (Tokuhisa *et al.* 1998; Ahlert *et al.* 2003; Rogalski *et al.* 2008; Liu *et al.* 2010). Taken together, *TSV3* might be involved in a protective mechanism under cold stress and the reduction of TSV3 would lead to a cold-sensitive chloroplast deficiency.

**Figure 9:**
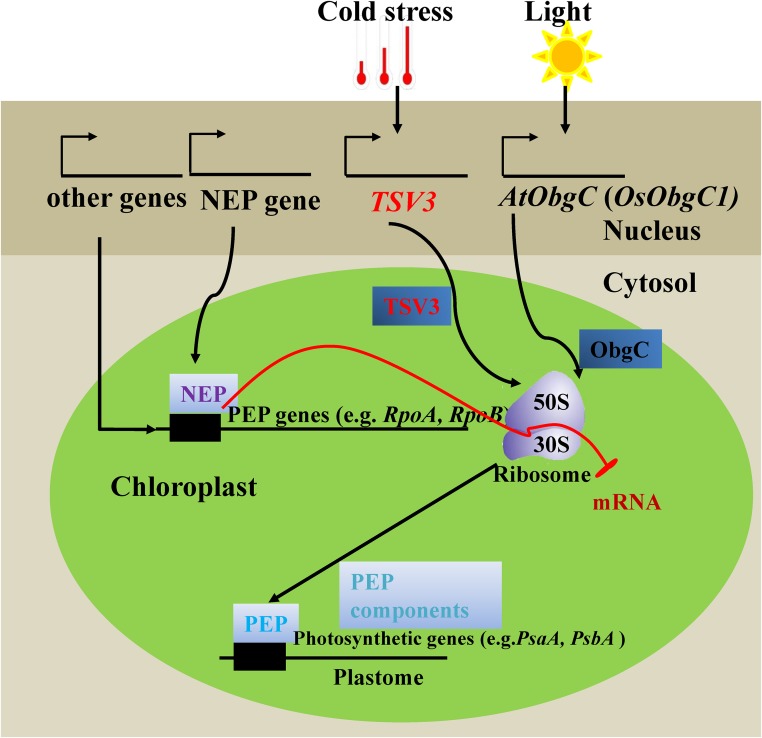
A functional model of *AtObgC* in *Arabidopsis* (Bang *et al.* 2012) and the possible model of *TSV3* in developing chloroplast. Two types of RNA polymerases (NEP and PEP) have been identified in higher plant chloroplasts. TSV3 interacts with the rice chloroplast 50S ribosome subunit, which functions in the translation of protein encoded by the chloroplast gene and also regulates the transcription of photosynthetic or some housekeeping genes by affecting PEP synthesis. In the absence of TSV3, PEP activity stays low, which couples biosynthesis of Chl and proteins, and is significantly reduced at the seedling stage under cold stress, leading to the virescent phenotype.

In conclusion, our data clearly indicated that *TSV3* was fundamentally involved in the biogenesis of plastid ribosomes under cold stress during chloroplast development in early leaves. A hypothetical model for *TSV3* function is shown in Figure 9. In this model, analogous to *AtObgC/OsObgC1* which is translocated into the chloroplast under light-induced conditions (Bang *et al.* 2012), TSV3 was translocated into the chloroplast under cold stress to interact with the chloroplast ribosomal 50S subunit to produce active PEP capable of transcribing photosynthetic or housekeeping genes. Gaining a better understanding of *TSV3* function and whether it might control expression of active PEP, according to cell type, developmental stage, or environmental conditions merits further investigation.

## Supplementary Material

Supplemental material is available online at www.g3journal.org/lookup/suppl/doi:10.1534/g3.117.300249/-/DC1.
