# Supplementary figures and images for: Rice TSV3 Encoding Obg-Like GTPase Protein Is Essential for Chloroplast Development During the Early Leaf Stage Under Cold Stress
Source: G3 (Bethesda). 2017 Nov 21;8(1):253–63. doi: 10.1534/g3.117.300249 (PMC5765353; doi:10.1534/g3.117.300249)

## Slide 1
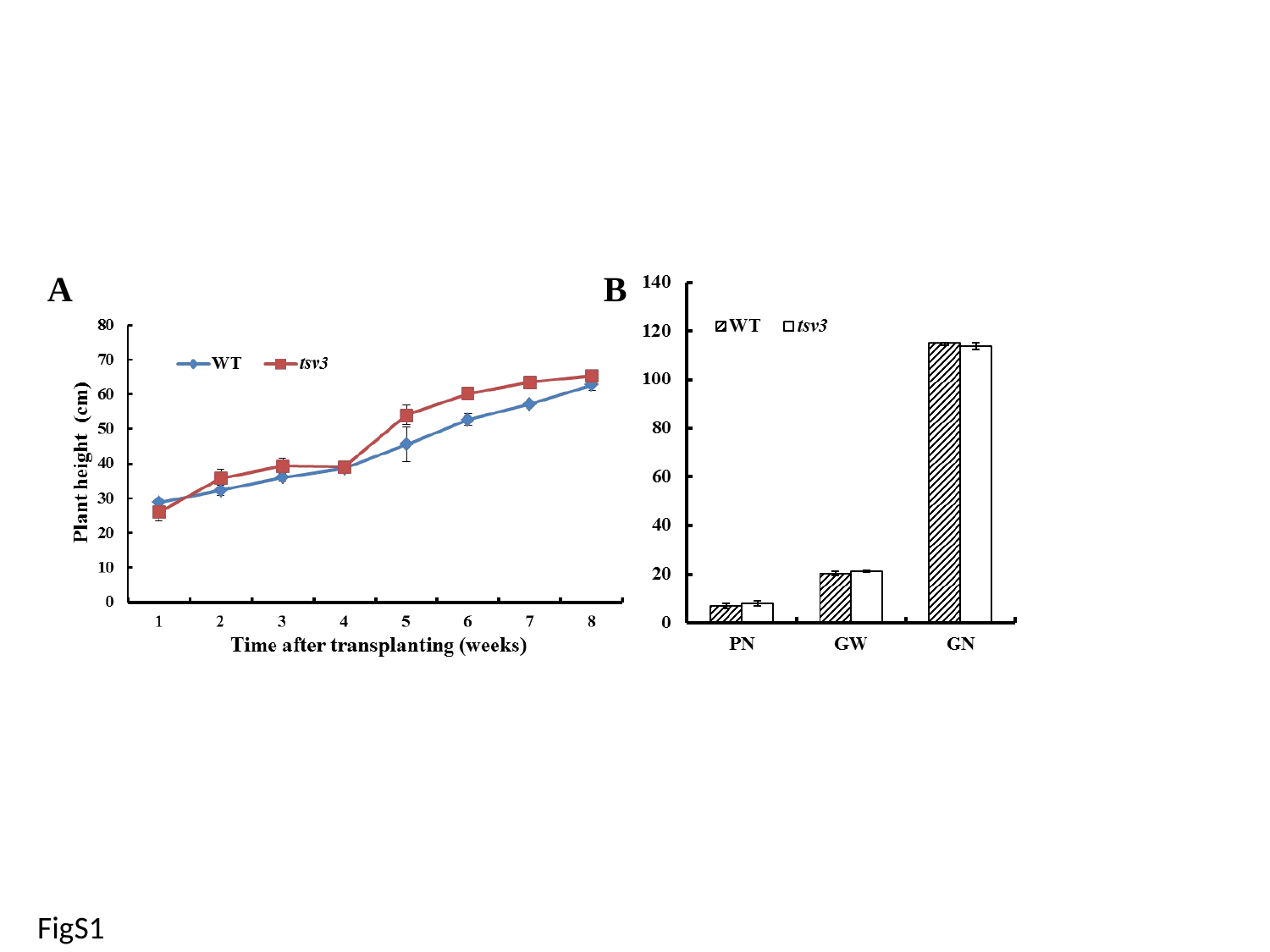

A B
FigS1

Supplement: Supplementary file 1 [file 253FigureS1.pptx]

## Slide 1
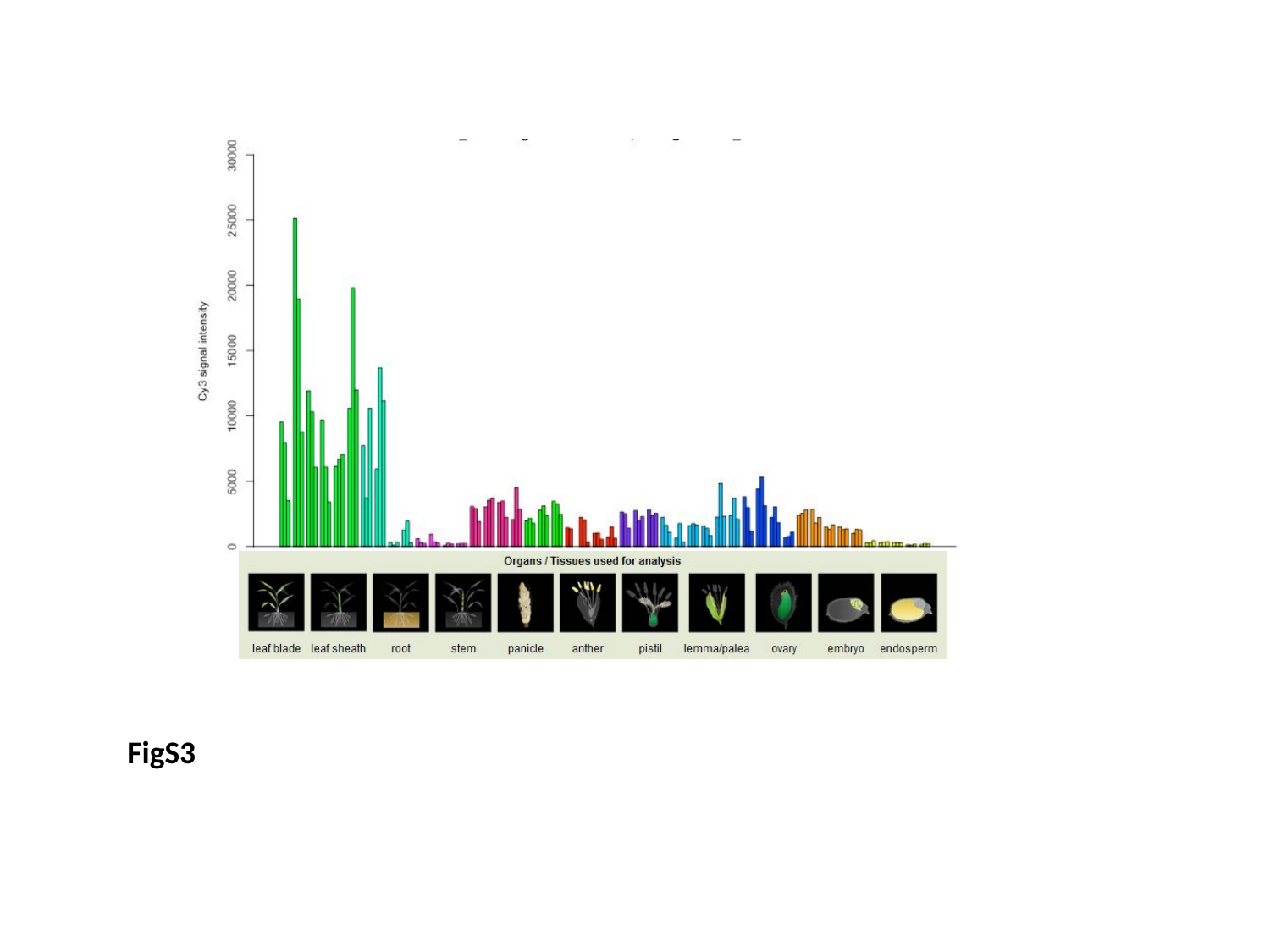

FigS3

Supplement: Supplementary file 3 [file 253FigureS3.pptx]

## Slide 1
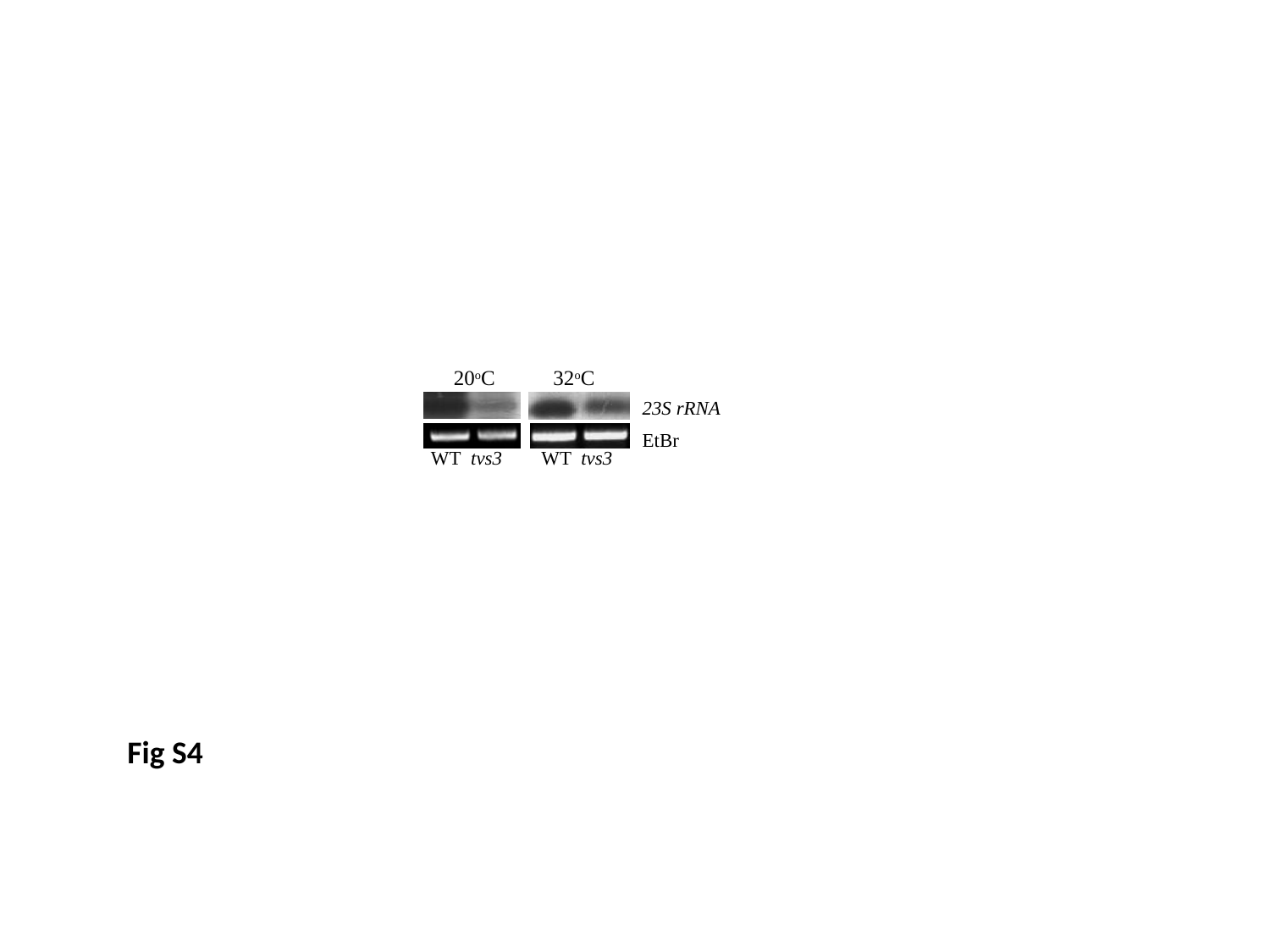

20oC 32oC
 23S rRNA
 EtBr
 WT tvs3 WT tvs3
Fig S4

Supplement: Supplementary file 4 [file 253FigureS4.pptx]
