# Supplementary material for: Rice TSV3 Encoding Obg-Like GTPase Protein Is Essential for Chloroplast Development During the Early Leaf Stage Under Cold Stress
Source: G3 (Bethesda). 2017 Nov 21;8(1):253–63. doi: 10.1534/g3.117.300249 (PMC5765353; doi:10.1534/g3.117.300249)
Supplement: Supplementary file 2 [file 253FigureS2.pptx]

## Slide 1
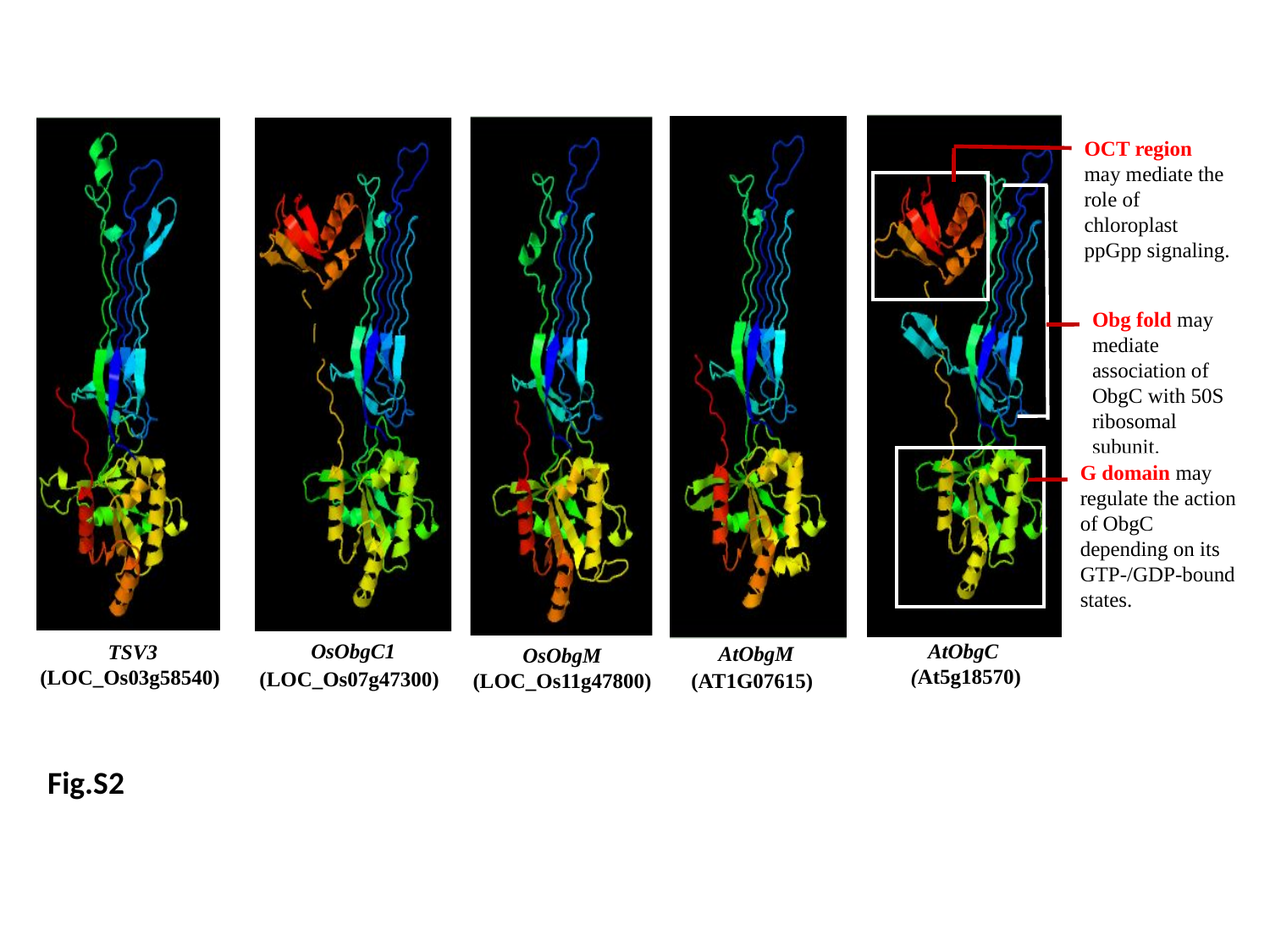

OCT region may mediate the role of chloroplast ppGpp signaling.
Obg fold may mediate association of ObgC with 50S ribosomal subunit.
G domain may regulate the action of ObgC depending on its GTP-/GDP-bound states.
 OsObgC1
(LOC_Os07g47300)
 AtObgM
(AT1G07615)
AtObgC
 (At5g18570)
 TSV3
(LOC_Os03g58540)
OsObgM
(LOC_Os11g47800)
Fig.S2
