# Supplementary material for: Rice TSV3 Encoding Obg-Like GTPase Protein Is Essential for Chloroplast Development During the Early Leaf Stage Under Cold Stress
Source: G3 (Bethesda). 2017 Nov 21;8(1):253–63. doi: 10.1534/g3.117.300249 (PMC5765353; doi:10.1534/g3.117.300249)
Supplement: Supplementary file 5 [file 253TableS1.doc]

**Supplemental Table S1.** PCR-based molecular markers designed for fine mapping *TSV3*.

| **No.** | **Marker** | **Forward sequence (5′→3′)** | **Reverse sequence (5′→3′)** |
| --- | --- | --- | --- |
|  | RM16 | CGCTAGGGCAGCATCTAAA | AACACAGCAGGTACGCGC |
|  | RM570 | GTTCTTCAACTCCCAGTGCG | TGACGATGTGGAAGAGCAAG |
| P1 | Chr03ID33003 | AAAATGGTGGCGTGATGTG | ACCACAAGGACAAGGTGATTAG |
| P2 | Chr03ID33307 | TGCCCAAAATGCCAAAAC | ATTGCCCACCAGTAGAATCATC |
| P3 | Chr03ID33318 | GATAGAGGACTTATGCGGATTAC | ACCTGCCTGTAACAGAATGATC |
| P4 | Chr03ID33320 | GCCTATCAAATCAACCTATGAACTC | TCTAACACCAATATAAAGCGGC |
| P5 | Chr03ID33343 | ATGGCAGTAGGATCTCATGTCAAC | CTGTTCCATTGCTCTCAAGGTATC |
| P6 | Chr03ID33386 | AAGAAGGAGCCCATCTCGGTC | CGAAGCGAAGAGGAGCAAG |
| P7 | Chr03ID33462 | CCTAGTCCTACAAACATAACGCAC | TCTTCTCTGCTCATGCAATCCA |
| P8 | Chr03ID34292 | TGCTGCTACTAGTATTTGTCCACC | AGGACTTGCTCAGCATCTTCAG |
| P9 | Chr03ID34393 | TCTACGTGTAACCAACACATCACC | CGATGAACGCTACTATCCAGAGG |
| P10 | Chr03ID35013 | CTCACTTTACAGGATGGGCAC | GCTGGGCAATCACAATACAC |
