# Supplementary material for: Rice TSV3 Encoding Obg-Like GTPase Protein Is Essential for Chloroplast Development During the Early Leaf Stage Under Cold Stress
Source: G3 (Bethesda). 2017 Nov 21;8(1):253–63. doi: 10.1534/g3.117.300249 (PMC5765353; doi:10.1534/g3.117.300249)
Supplement: Supplementary file 6 [file 253TableS2.doc]

**Supplemental Table S2**. Markers designed for real-time RT-PCR.

| **Gene** | **Forward primer (5′→3′)** | **Reverse primer (5′→3′)** |
| --- | --- | --- |
| *TSV3* | CACGAGAAGGCCCGATACT | GGATGAGCCAAATCAGCAAG |
| *Rps7* | gccaaaatccattccaattc | ggagatgtacacgaggagattg |
| *Rps20* | CACGCTCTTCTCCCTCTCCT' | GTAGGAGGCGGACAGGCG |
| *16S rRNA* | CCGTTGGTGTTCTTTCCGAT | TTCAAGTCCGCCGTCAAATC |
| *23S rRNA* | TGTGGGCGTTAGAGCATTGAG | CACTTGGCTACCCAGCGTTTA |
| *Rpl21* | AAGAAGAGGAGGCTGCGGT | GACATTGGCGCCTTTCAGC |
| *V2* | GAGGAGTTCCTCACGATGAT | AGCATCAATGATAGACTCC |
| *RNRL* | gttagatgcttcactacacag | gtaccattgccaacatggcaac |
| *RNRS* | gccaaaatccattccaattc | ggagatgtacacgaggagattg |
| *LhcpII* | GAAGAAGATCAAGAACGGCC | TTGCCGGGGACGAAGTTGGT |
| *RbcS* | TCCGCTGAGTTTTGGCTATTT | GGACTTGAGCCCTGGAAGG |
| *FtsZ* | AAAGGACATAACCTTGCAAG | AGTTTTCCTATTGAACCGTG |
| *OsPOLP1* | ACCGGTGCTTTCAGGCTTGG | GCTGACTGATAATCACACG |
| *OsRpoTp* | AAGCAGACAGTGATGACATC | ATCACATGCATGCACCCAAA |
| *RpoB* | TTTGGTTTCGATGTGCA | TATGGTCTAATTCCGAGCGGT |
| *PsaA* | GCGAGCAAATAAAACACCTTTC | GTACCAGCTTAACGTGGGGAG |
| *PsbA* | CCCTCATTAGCAGATTCGTTTT | ATGATTGTATTCCAGGCAGAGC |
| *RbcL* | CTTGGCAGCATTCCGAGTAA | ACAACGGGCTCGATGTGATA |
| *RpoA* | GTGGAAGTGTGTTGAATCAA | TCTCTCTTGATCCGTAACTC |
| *CAO1* | GATCCATACCCGATCGACAT | CGAGAGACATCCGGTAGAGC |
| *PORA* | TGTACTGGAGCTGGAACAACAA | GAGCACAGCAAAATCCTAGACG |
| *YGL1* | CAGTCTCCAATGGCCACCT | TGCTTTCATCAGTGGCTGGT |
| *Cab1R* | AGATGGGTTTAGTGCGACGAG | TTTGGGATCGAGGGAGTATTT |
| *Actin* | AGGAAGGCTGGAAGAGGACC | CGGGAAATTGTGAGGGACAT |
