# Supplementary material for: Rice TSV3 Encoding Obg-Like GTPase Protein Is Essential for Chloroplast Development During the Early Leaf Stage Under Cold Stress
Source: G3 (Bethesda). 2017 Nov 21;8(1):253–63. doi: 10.1534/g3.117.300249 (PMC5765353; doi:10.1534/g3.117.300249)
Supplement: Supplementary file 7 [file 253FileS1.docx]

**Supplemental Figure 1** Characterization of the WT and *tsv3* mutant plants grown under field conditions in Shanghai, China in 2010. **A** Changes in plant height from transplanting to heading. **B** Comparison of yield-related traits among WT and *tsv3* mutant plants. PN, panicle number per plant; GW: 1000-grain weight (g); GN, grain number per panicle.

**Supplemental Figure 2** Predicted 3D structures of TSV3 and homologous rice proteins. Data were predicted using the Phyre 2 server (http://www.sbg.bio.ic.ac.uk/phyre2/html/page.cgi?id=index). Functions of the three ObgC domains in AtObgC are cited from Bang et al. (2012).

**Supplemental Figure 3** Expression patterns of *TSV3* (*LOC_Os03g58540*). Data are cited from the rice expression profile database, RiceXPro (http://ricexpro.dna.affrc.go.jp/category-select.php).

**Supplemental Figure 4** RNA blot analysis of the chloroplast 23S ribosomal RNA. Five micrograms of total RNA was isolated from the 3-leaf-stage seedlings of WT and *tsv3* mutant grown at 20°C and 32 °C was extracted. RNA gel blot analysis was performed as described previously (Chi et al. 2014). In addition, the 25S rRNA stained with ethidium bromide (EtBr) is shown as a loading control.

**Table legends**

**Supplemental Table 1** PCR-based molecular markers designed for fine mapping *TSV3*.

**Supplemental Table 2** Markers designed for real-time qPCR.
